# A Review of Aphid Parasitoids, with an Identification Key to the Genera of Economic Importance

**DOI:** 10.3390/insects16070648

**Published:** 2025-06-20

**Authors:** Mar Ferrer-Suay, Marc Barreda, Ehsan Rakhshani, Eugenia Rodrigo, Jesús Selfa, Andrew Polaszek

**Affiliations:** 1 Departament de Zoologia, Facultat de Ciències Biològiques, Universitat de València, Campus de Burjassot-Paterna, Bloc B, Dr. Moliner 50, 46100 Burjassot, Spain; 2 Department of Plant Protection, College of Agriculture, University of Zabol, Zabol 98615-538, Iran; rakhshani@uoz.ac.ir; 3 Instituto Agroforestal del Mediterráneo, Universitat Politècnica de Valencia, Camino de Vera s/n, 46022 Valencia, Spain; erodrigo@eaf.upv.es; 4 Science: Research, Natural History Museum, London SW7 5BD, UK; a.polaszek@nhm.ac.uk

**Keywords:** Aphididae, Aphidiinae, Aphidoidea, Braconidae, Chalcidoidea, hyperparasitoids, secondary parasitoids, biological control programs, biocontrol

## Abstract

Agriculture plays an essential role in meeting global food demand, and so effective pest control is crucial. Aphids (Hemiptera: Aphididae) are a significant problem for many crops worldwide, and cause crop losses. They are considered one of the most important groups of pests in the world. Aphid parasitoids are very important as biological agents, both for their effectiveness and their host range selectivity. This study provides a review of these groups of parasitoids and their possible role and use in aphid biological control. Two groups of primary parasitoids, Aphelinidae and Braconidae: Aphidiinae, are the main groups that can be used in the biological control of aphids. There is also a diversity of hyperparasitoids whose hosts are these primary parasitoids and therefore are likely to be detrimental to the biological control programs: Megaspilidae in the superfamily Ceraphronoidea, the following Chalcidoidea families: Encyrtidae, Eulophidae, Moranilidae, Pteromalidae, and Signiphoridae, and Cynipoidea: Figitidae: Charipinae. Records of Mymaridae emerging from aphids, while assumed to be obligate egg-parasitoids, are discussed. Dichotomous keys are provided to identify the superfamilies, families and most important genera associated with aphids.

## Simple Summary

Agriculture relies on effective pest control to meet global food demands, with aphids being one of the most damaging pest groups worldwide. Hymenopteran aphid parasitoids play a key role as biological control agents due to their efficiency and selectivity. The two main primary parasitoid groups treated here are Aphelinidae (Chalcidoidea) and Braconidae (Ichneumonoidea). Additionally, various hyperparasitoid families, such as Ceraphronoidea: Megaspilidae; Chalcidoidea: Encyrtidae, Eulophidae, Moranilidae, Pteromalidae and Signiphoridae, and Cynipoidea: Figitidae: Charipinae, are included because of their potential to undermine pest control efforts by attacking primary parasitoids. The study provides dichotomous keys to identify the superfamilies, families and economically important genera associated with aphids.

## 1. Introduction

In the coming decades, the global spread of pests will continue to be a major problem for agriculture. If the current trends in pest spread persist, many of the world’s leading crop-producing nations are likely to be overwhelmed by pests by the middle of the century [1]. Under climate change factors pest infestation and crop damage are becoming more likely scenarios, and we then face a higher risk of significant economic losses and a challenge to human food security [2,3,4].

Aphididae (aphids) are hemipteran insects belonging to the superfamily Aphidoidea. They belong to the suborder Sternorrhyncha, along with Aleyrodoidea (whiteflies), Psylloidea (jumping plant lice), and Coccomorpha (scale insects). Aphidoidea has three extant families: Adelgidae (adelgids), Phylloxeridae (phylloxerids) and Aphididae. Most species are found in the family Aphididae. Around 450 species have taken advantage of the agricultural environment, and of these about 100 have become important pests that incur significant economic costs [5]. Aphids reduce agricultural yields in several ways. They inflict damage through direct feeding, and indirectly by transmitting pathogens to plants [6]. For aphid biological control a large number of organisms can be useful, mainly arthropods that control other arthropods (pests) essentially through two processes: predation and parasitoidism. Insect parasitoids are organisms that develop on or in a single insect host, eventually killing it [7]. During their immature phase, they feed on the body fluids and organs of the host. Hymenoptera and Diptera are the most prominent groups of parasitoids, and specialize in the choice of their host. In the case of aphid parasitoids, all are Hymenoptera. Specificity in the parasitoid-host interaction has led to the success of biological control using parasitoids [8].

## 2. Materials and Methods

To conduct this study, an extensive search was performed for scientific literature regarding the association of aphid parasitoids, utilizing various academic search engines. Records of Chalcidoidea as aphid parasitoids or hyperparasitoids downloaded from a non-public version of the Universal Chalcidoidea Database [9]. Many databases, however, are known to contain misidentifications, and these are often perpetuated and repeated without checking. For this reason, where records were from families for which we as authors have limited expertise, we consulted with the foremost experts, notably Dr John Noyes, Natural History Museum, London (NHMUK) for the records of Encyrtidae genera. We have therefore been conservative in our inclusion of genera, for which the criteria for inclusion are discussed below, under each family.

The taxonomic part of this study includes examination of various genera in each of the study groups, approximately about 3800 specimens from Aphidiinae (Braconidae), about 22,000 specimens from Chalcidoidea (five families), about 100 specimens from Megaspilidae (Ceraphronoidea), and more than 10,000 specimens from Charipinae (Figitidae). Most of the specimens examined were collected (or received) over the past 40 years by the authors. When attempting to rear aphid parasitoids, infested sections of host plants were cut and, after removing other insects, transported to the laboratory. They were kept at room temperature in permeable containers until the emergence of the parasitoids. To minimize hyperparasitoids inside the containers, visits were made daily, and any wasps that emerged were collected using an aspirator and stored in microtubes containing 75% or 95% ethanol. In cases where parasitized aphids had already been mummified, they were kept separately in gelatin capsules under laboratory conditions until emergence of the parasitoids/hyperparasitoids. These series of examined materials are deposited in the collections of the Department of Plant Protection, University of Zabol, Iran (DPPZ); Insect Collection Natural History Museum, London, UK (NHMUK), and in the Zoology collection of the University of Valencia, Spain. Additionally, part of the study was based on specimens housed in the Canadian National Collection of Insects, Arachnids, and Nematodes (CNC).

For figure preparation in most cases dissected fore wings were mounted in Canada balsam on microscope slides. For the Aphidiinae wasps, female specimens were dissected, and body parts that included diagnostic characteristics (mesonotum, propodeum, petiole, genitalia) were slide mounted, as mentioned above. For image capture, specimens were studied with a stereoscopic microscope (Optika ZSM-2, Ponteranica, Italy) and images were taken with a Leica M80 stereo microscope with an attached IC90E camera (Wetzlar, Germany). Fore wing images were taken with an Olympus BX63 compound microscope (Tokyo, Japan)using either bright field illumination or Nomarski differential interference contrast (DIC). Line drawings were generated by tracing the characters on the digital photographs from the slides using Adobe Illustrator CS6. Morphological terminology used for diagnostics in the identification keys follows Sharkey and Wharton [10]—for Aphidiinae (Braconidae), Ferrer-Suay et al. [11]—for Charipinae, and Gibson [12]—for Chalcidoidea.

**Figure 1:**
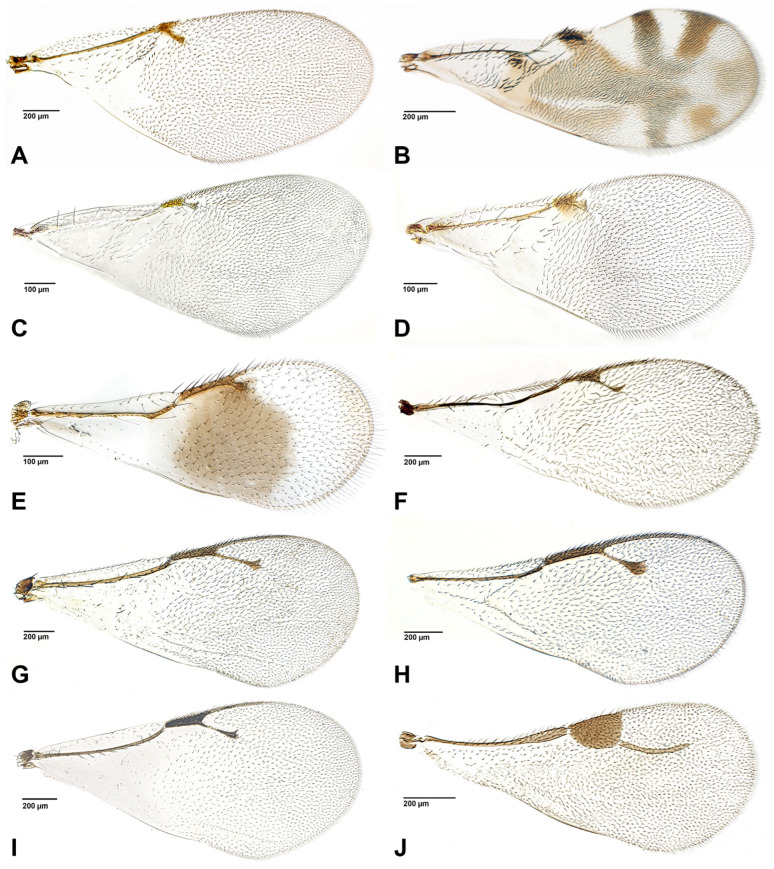
Fore wings of parasitoid genera associated with aphids. (**A**). *Bothriothorax* sp. (Encyrtidae); (**B**). *Cerapteroceroides* sp. (Encyrtidae); (**C**). *Syrphophagus* sp. (Encyrtidae); (**D**). *Tassonia* sp. (Encyrtidae); (**E**). *Moranila* sp. (Moranilidae); (**F**). *Asaphes* sp. (Chalcidoidea *incertae sedis*); (**G**). *Euneura* sp. (Pteromalidae); (**H**). *Coruna* sp. (Pteromalidae); (**I**). *Pachyneuron* sp. (Pteromalidae); (**J**). *Dendrocerus* sp. (Megaspilidae).

**Figure 2:**
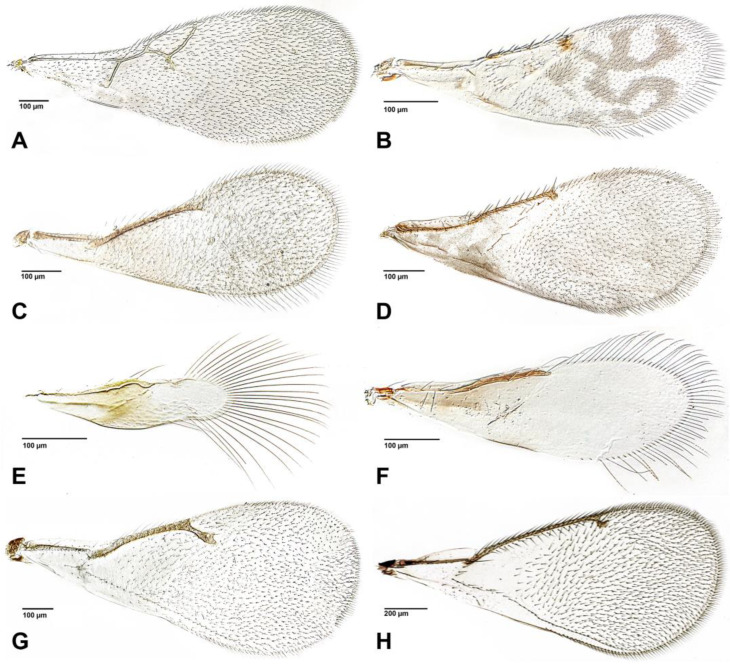
Fore wings of parasitoids associated with aphids. (**A**). *Alloxysta* sp. (Figitidae); (**B**). *Marietta* sp. (Aphelinidae); (**C**). *Encarsia* sp. (Aphelinidae); (**D**). *Aphelinus* sp. (Aphelinidae); (**E**). *Signiphora* sp. (Signiphoridae); (**F**). *Chartocerus* sp. (Signiphoridae); (**G**). *Tetrastichus* sp. (Eulophidae); (**H**). *Pediobius* sp. (Eulophidae).

**Figure 3:**
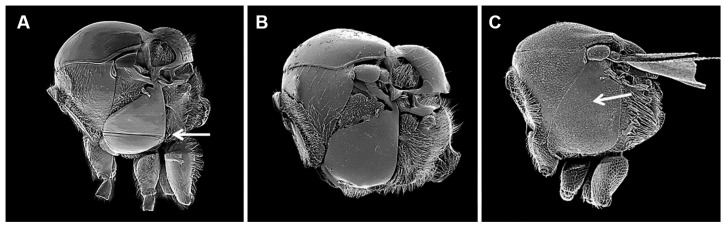
Mesosoma in lateral view Charipinae genera (Figitidae) associated with aphids: (**A**). *Phaenoglyphis* sp. (arrow show mesopleural sulcus); (**B**). *Alloxysta* sp.; (**C**). *Lytoxysta* sp. (arrow show the lack of mesopleural triangle).

**Figure 4:**
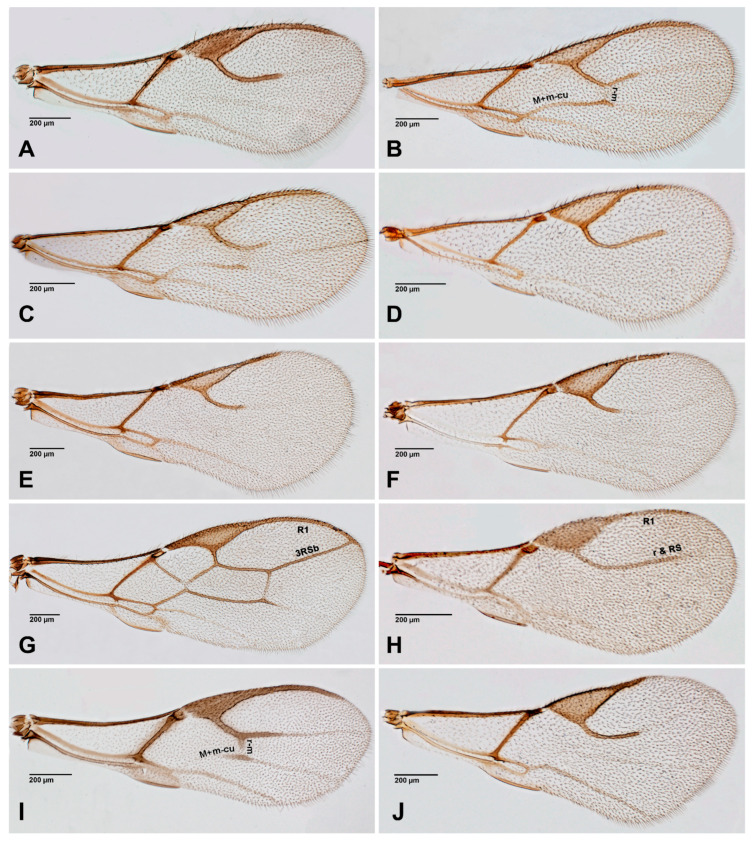
Aphidiinae fore wings, females. (**A**). *Adialytus thelaxis*; (**B**). *Aphidius colemani*; (**C**). *Aphidius matricariae*; (**D**). *Binodoxys angelicae*; (**E**). *Diaeretiella rapae*; (**F**). *Diaeretus leucopterus*; (**G**). *Ephedrus plagiator*; (**H**). *Lipolexis gracilis*; (**I**). *Lysiphlebus cardui*; (**J**). *Monoctonia vesicarii*.

**Figure 5:**
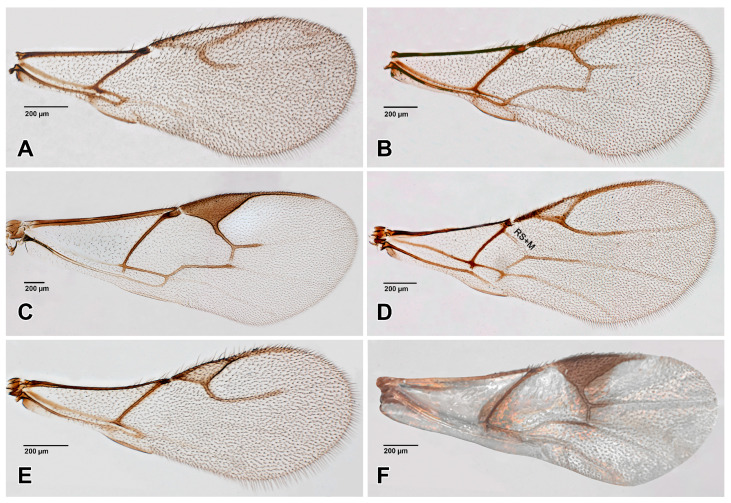
Aphidiinae, fore wings. (**A**). *Monoctonus cerasi*; (**B**). *Monoctonus crepidis*; (**C**). *Pauesia abietis*; (**D**). *Praon* sp.; (**E**). *Trioxys* sp.; (**F**). *Xenostigmus bifasicatus*.

**Figure 6:**
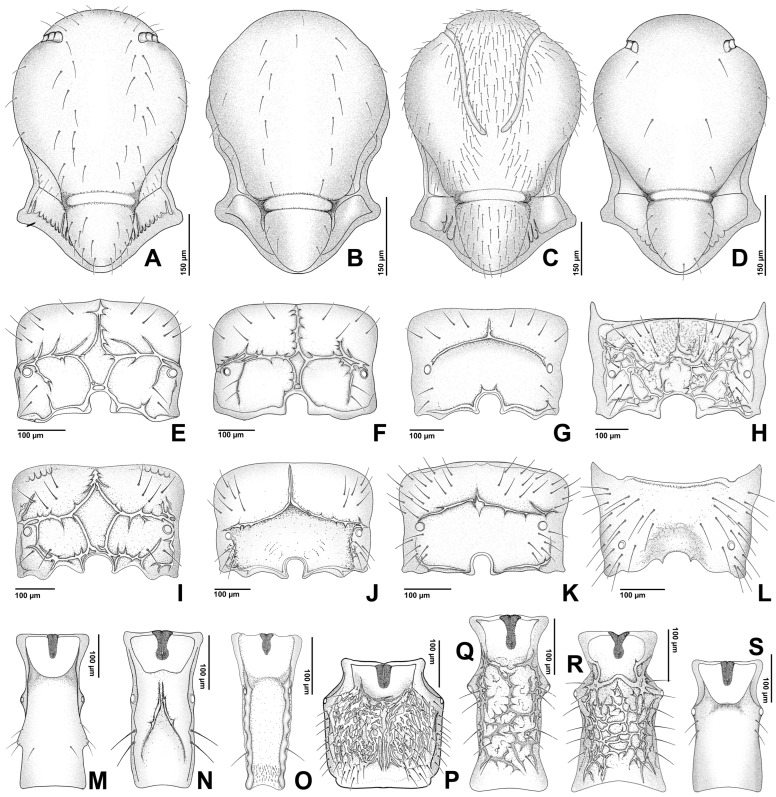
(**A**–**D**). Mesonotum; (**E**–**L**). Propodeum; (**M**–**S**). Petiole. (**A**,**E**). *Aphidius matricariae*; (**B**,**G**). *Diaeretus leucopterus*; (**C**). *Praon barbatum*; (**D**,**S**). *Trioxys pallidus*; (**F**). *Diaeretiella rapae*; (**H**,**P**). *Monoctonis vesicarii*; (**I**,**Q**). *Monoctonus crepidis*; (**J**). *Pauesia abietis*; (**K**). *Pauesia silana*; (**L**). *Xenostigmus bifasciatus*; (**M**). *Binodoxys angelicae*; (**N**). *Lipolexis gracilis*; (**O**). *Lipolexis oregmae*; (**R**). *Monoctonus cerasi*.

**Figure 7:**
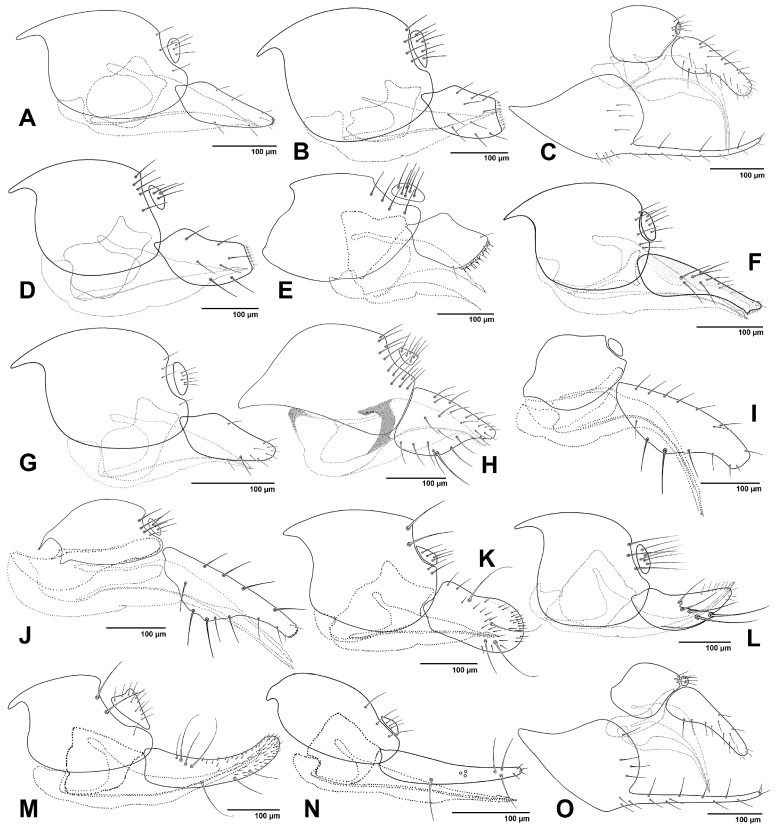
Female genitalia, lateral view. (**A**). *Adialytus thelaxis*; (**B**). *Aphidius matricariae*; (**C**). *Binodoxys angelicae*; (**D**). *Diaeretiella rapae*; (**E**). *Diaeretus leucopterus*; (**F**). *Lipolexis gracilis*; (**G**). *Lysiphlebus cardui*; (**H**). *Monoctonia vesicarii*; (**I**). *Monoctonus crepidis*; (**J**). *Monoctonus cerasi*; (**K**). *Pauesia abietis*; (**L**). *Pauesia hazratbalensis*; (**M**). *Pauesia picta*; (**N**). *Pauesia unilachni*; (**O**). *Trioxys pallidus*.

The figures included in this study illustrate key diagnostic characters of aphid-associated parasitoids. Figure 1 and Figure 2 present the fore wings of representative genera across multiple families, highlighting intergeneric variation in venation patterns. Figure 3 focuses on mesosoma morphology in Charipinae, emphasizing features such as the mesopleural sulcus and the presence or absence of the mesopleural triangle. Figure 4 and Figure 5 detail the fore wings of Aphidiinae species. Figure 6 compares the mesonotum, propodeum, and petiole across selected Aphidiinae, while Figure 7 illustrates the lateral view of female genitalia, aiding in species-level differentiation.

## 3. Results

### 3.1. Hymenoptera Families Associated with Aphids

#### 3.1.1. Primary Parasitoids

Chalcidoidea

Aphelinidae (Figure 2C,D)

Most aphelinids are primary parasites of stenorrhynchous hemipterans (Aleyrodoidea, Aphidoidea, Coccoidea, and Psylloidea), all groups that can become important pests [13]; a few are known to develop in other hosts such as Orthoptera and Lepidoptera eggs, and Diptera pupae [14]. In some genera, male larvae are hyperparasitoids, while female larvae are always primary parasitoids [15,16,17]. Aphelinidae has 43 recognized genera, and about 1120 described species [9]. Within these genera, only three are primary parasitoids of aphids: all species of *Aphelinus* Dalman and *Protaphelinus* Mackauer and a few species of *Encarsia* Förster. Hyperparasitoid aphelinids associated with aphids are restricted to the genus *Marietta* Motschulsky [9].

*Aphelinus* species are primary parasitoids of aphids, with very few reliable records from other hosts. Collectively they have a very wide host range, of more than 40 aphid species in a broad range of genera [18]. In some cases, it has been possible to study the effectiveness of species of this genus as parasitoids and their effectiveness in controlling aphid populations [19,20]. This has meant that some species of this genus have been, and are, used as biological control agents, such as the successful use of *Aphelinus mali* (Haldeman) for the biological control of *Eriosoma lanigerum* (Hausmann), the apple woolly aphid [21]. Currently, ways to implement the use of parasitoids, and minimise the damage by pesticides, continue to be studied [21]. This is done through the release of species that are commercially produced such as *Aphelinus abdominalis* (Dalman) [22], or the attraction of *Aphelinus* by synthetic semiochemicals [23].

*Protaphelinus* species attack aphids in the genus *Pemphigus* [18,24]. The primary hosts of *Pemphigus* species are *Populus* (poplars), on which they cause galls on leaves and stems. Its secondary hosts include Asteraceae and Apiaceae, while some *Pemphigus* (Mackauer) species remain on a single host throughout the year [24].

Finally, the genus *Encarsia*, with nearly 500 species is the most speciose genus in this family [9]. Females of most species parasitize whiteflies (Aleyrodoidea) and scale insects of the family Diaspididae, with males being either hyperarasitoids of conspecific females, or those of other species, or even other genera [17]. The *Encarsia flavoscutellum* (Zehntner) species-group consists, as far as is known, of primary parasitoids of aphids in the subfamily Hormaphidinae. Immatures of this subfamily very strongly resemble whitefly nymphs or puparia [25]. Despite the great importance that *Encarsia* species have in the biological control of other pests such as whiteflies and armored scale insects, they are of minor importance for aphid control due to the paucity of aphid-associated species. However, two studies refer to the possible benefits of the presence of *Encarsia flavoscutellum* Zehntner in sugarcane crops due to control of the aphid *Ceratovacuna lanigera* Zehntner [26,27].

Mymaridae

Mymarids are almost exclusively primary egg-parasitoids. Despite the documented records of the emergence of these wasps from aphids [9], we cannot confidently verify the occurrence of parasitoidism or hyperparasitoidism of aphids by mymarids (e.g., *Polynema* Haliday). The specimens emerging during rearing are likely to have been parasitoids of the eggs of other insects, such as leafhoppers, which are often laid inconspicuously within plant tissue.

Ichneumonoidea

Braconidae (Aphidiinae) (Figure 4, Figure 5, Figure 6 and Figure 7)

The family Braconidae (Hymenoptera: Ichneumonoidea) is a very large family of Hymenoptera parasitoids that has around 22,000 described species belonging to 1250 genera [28]. Within this large family, there are 41 subfamilies [29], of which only Aphidiinae are aphid parasitoids [30]. This subfamily makes up a small group of about 500 species grouped in 38 genera worldwide, distributed mainly in the Holarctic region [31]. Due to the great host specificity of this subfamily, all species have an impact on aphid populations, although only some of these have been mass produced for commercial purposes. Boivin et al. [32] recorded some of the species that are commercially produced: *Aphidius colemani* Viereck, *A. ervi* Haliday, *A. gifuensis* Ashmead, *A. matricariae* Haliday, *A. urticae* Haliday, *Ephedrus cerasicola* Stary, *Lysiphlebus fabarum* (Marshall), *L. testaceipes* (Cresson) and *Praon volucre* (Haliday). In more recent articles, this group of parasitoids continues to be investigated, better understanding parasitoid-aphid relationships, or finding new species that can be commercialized and mass produced [33,34]. However, there are still many important aphid parasitoids for which almost no biological data are available [33].

#### 3.1.2. Secondary Parasitoids

Ceraphronoidea

Megaspilidae (Figure 1J)

Megaspilidae (Hymenoptera: Ceraphronoidea) is one of the least studied parasitoid families, so the biology of many of its species is still unknown [35]. It contains 355 species grouped into 13 genera [36]. The family appears to comprise mainly Diptera parasitoids, although there are many exceptions. Many species are primary ectoparasitoids that attack Diptera, Neuroptera, Coleoptera and Mecoptera. However, there are also some that are pseudohyperparasitoids of aphids [35], mainly in the genus *Dendrocerus*. By pseudohyperparasitoids we mean that *Dendrocerus* larvae develop when the aphid is in the mummy stage and the aphidiine is in the pupa stage, thus parasitoidism of aphids by *Dendrocerus* species is indirect. Some species are primary parasitoids of other species of predatory insects such as the larvae or pupae of aphidophagous hoverflies (Syrphidae), lacewings (Chrysopidae), or ladybird beetles (Coccinellidae) [37].

*Dendrocerus carpenteri* (Curtis) is a cosmopolitan species that has been widely studied compared to most species in the family. It is a solitary ecto-hyperparasitoid of aphidiines, that attacks the prepupal and pupal stages of the primary parasitoid once the aphid is mummified [38,39]. Genera such as *Praon* that pupate in a cocoon under the aphid mummy are less accessible than other genera such as *Aphidius*, *Lysiphlebus*, or *Ephedrus* that pupate inside the aphid [39].

Chalcidoidea

Aphelinidae (Figure 2B)

Species of *Marietta* are hyperparasitoids, mainly of scale insects (Coccomorpha), but can also be hyperparasitoids of aphids. They have been recorded from *Aphis gossypii* Glover and *A. craccivora* Koch [9]. While hyperparasitoids are usually considered detrimental to natural cropping systems, their possible stabilizing effect on host-parasitoid population swings has been discussed [40]. A single male *Encarsia* has been reliably reared from a *Paoliella* aphid in East Africa (A. Polaszek, unpublished observation). It is very likely to have been a hyperparasitoid.

Encyrtidae (Figure 1A–D)

Encyrtidae includes around 3735 species described in 460 genera [9]. These are grouped into two subfamilies: Encyrtinae and Tetracneminae. The family is of great importance in biocontrol, especially of mealybugs and other scale insects [9]. In the case of aphids, they act mostly, or exclusively, as hyperparasitoids. *Syrphophagus* Ashmead, is a good representative of aphid hyperparasitoidism [41]. *Syrphophagus aphidivorus* (Mayr) has been studied in some detail and is recorded as having a dual behavior in the parasitoidism of aphidiines. It not only oviposits when the primary parasitoid is in the prepupa or pupa state, but it can also do so when it is in the larval stage and the aphid is not yet mummified. This may give it an advantage over other hyperparasitoids in the struggle to find viable hosts [42,43]. In addition to *Syrphophagus*, aphid hyperparasitoidism has been attributed to *Leptomastidea* Mercet. *Leptomastidea bifasciata* (Mayr) has been recorded as a secondary parasitoid of the aphid *Cinara juniperi* (De Geer) which attacks *Juniperus* trees [44]. We consider this record as erroneous or at least very unlikely, as *Leptomastidea* species are parasitoids of mealybugs (Pseudococcidae). A range of other encyrtids have been recorded as aphid hyperparasitoids; here we include what we consider to be the most reliable records, including *Bothriothorax* Mayr, *Ceraptoroceroides* Grissell, and *Tassonia* Ashmead [9]. Of these, *Bothriothorax* and *Ceraptoroceroides* are either very rarely parasitoids of aphids, or possibly not at all. Published records of *Blastothrix* and *Microterys* from aphids [9] are considered here as erroneous.

Eulophidae (Figure 2G,H)

Eulophidae includes 324 genera, and approximately 5300 species distributed in 4 subfamilies [9]. Most species are primary parasitoids of concealed larvae, especially leaf miners. The best-studied species attack dipterans and lepidopterans, but many species are parasitoids of other insects that live in a similar way [9]. Although species within the family are known for wide host ranges and reproductive strategies, there is not much information on their relationship with aphids [45]. An example of aphid hyperparasitoidism is the genus *Tetrastichus* Haliday, which was found in mummies of *Macrosiphum rosae* (L.) [46] and *Pauesia antennata* (Mukerji) [47]. It has recently been recorded that *Tamarixia* species could be primary or secondary parasitoids of aphids [48], although their role is not very clear.On the other hand, aphid parasitoidism has also been recorded in *Oomyzus scaposus* (Thomson) found in the mummies of *Brachycaudus helichrysi* (Kaltenbach), being normally a hyperparasitoid of coccinellids [49]. Without the specific methods outlined in that study, it is challenging to assume an association with the aphids, instead of with the eggs of coccinellid aphid predators laid inside curled leaves.

Moranilidae (Figure 1E)

This family was recently elevated from being a subfamily (Moranilinae) of Pteromalidae [50]. Species of *Moranila* are mostly endoparasitoids or egg/larval predators of Coccoidea (Hemiptera), occasionally hyperparasitoids through other Chalcidoidea within Aphididae [50]. *Moranila comperei* (Ashmead) has been recorded from aphids several times [9].

Pteromalidae (Figure 1G–I)

Pteromalidae formally included almost 600 genera and 3500 species, but a recent reassessment of the superfamily erected 23 new families that were formerly subfamilies of Pteromalidae [50], including Moranilidae, above. Within the current Pteromalidae aphid hyperparasitoidism is restricted to Pteromalinae with *Pachyneuron* species standing out as common parasitoids of some primary parasitoids of aphids such as *Lysiphlebus testaceipes* and *Diaeretiella rapae* (M’Intosh) [51]. *Pachyneuron aphidis* Walker is the most common hyperparasitoid of aphids. Other less studied genera also occur, such as *Euneura* and *Coruna* [52]. *Asaphes* species are also extremely common hyperparasitoids of aphids [9,52]. All of them are solitary ectohyperparasitoids of aphidiines and aphelinids that lay their eggs on top of the prepupa or pupa of the primary parasitoids. However, in the case of *Praon*, the hyperparasitoid develops outside the mummified aphid. *Euneura* Walker is one of the few hyperparasitoid genera developing in *Pauesia* spp. [53] (Aphidiinae). The current position of *Asaphes* within the subfamily Asaphesinae, is currently *incertae sedis* within Chalcidoidea [50].

Signiphoridae (Figure 2E,F)

Signiphoridae is currently a very small family of just 79 species described worldwide grouped into 4 genera: *Signiphora* Ashmead, *Thysanus* Walker, *Chartocerus* Motschulsky and *Clytina* Erdös [9,54]. They are mostly primary parasitoids or hyperparasitoids of sternorrhynchous Hemiptera, and are closely related to Azotidae and Aphelinidae [54]. Some studies have included observations of *Chartocerus* species parasitising *Aphidius* [55] or *Signiphora* attacking *Lipolexis oregmae* (Gahan) and *Lysiphlebus testaceipes* [56].

Cynipoidea

Figitidae (Charipinae) (Figure 3)

Figitidae includes around 1400 species described in 132 genera [57]. They are characterized by being parasitoids of the larvae of other insects, mainly of the order Diptera (Cyclorrapha) except for the subfamily Charipinae whose species are hyperparasitoids of Hymenoptera [58]. Currently, the Charipinae is divided into 8 genera: *Alloxysta* Förster, *Apocharips* Fergusson, *Dilapothor* Paretas-Martínez & Pujade-Villar, *Dilyta* Förster, *Lobopterocharips* Paretas-Martínez & Pujade-Villar, *Lytoxysta* Kieffer, *Phaenoglyphis* Förster, and *Thoreauana* Girault [11]. The oviposition behavior of *Alloxysta victrix* (Westwood) in the *Medicago sativa* L./*Acyrthosiphon pisum* Harris/*Aphidius smithi* Sharma & Subba Raocomplex was studied in detail [59]. The hyperparasitoid prefers aphids in the second or third stage of development in which it oviposits, and the stages of development occur in the hemocoel of the parasitized aphid still alive, including its embryonated eggs. We recently established the relation between *Alloxysta consobrina* (Zetterstedt)—*Diaretiella rapae* (McIntosh)*—Brevicoryne brassicae* (L.) in Valencia (Spain) on zucchini (unpublished data).

### 3.2. Aphid Parasitoid Key

Below is a key for the identification of families, subfamilies and genera of economic importance associated with aphids:

1 Brachypterous, wings hardly developed. ..............................*Aphelinus* (Aphelinidae)

— Fully winged, wings at least as long as body. ..............................................................2

2 Fore wing with costal vein, ending in a large triangular (Figure 4 and Figure 5 (Aphidiinae) or semicircular, pterostigma Figure 1J *Dendrocerus*)). .................................................3

— Fore wing without pterostigma (Figure 1A–I and Figure 2A–H). ........................................4

3 Metasoma cylindrical or depressed dorso-ventrally, with first apparent tergite very large, at least as wide as long, or longer than the following tergites combined. Antennal scape more than twice as long as wide.....................................................................................................................CERAPHRONOIDEA (Megaspilidae) *Dendrocerus*

— Metasoma compressed laterally, with the first tergite approximately the same length or shorter than the other metasomal tergites. Antennal scape at most twice as long as wide.................ICHNEUMONOIDEA (Braconidae: Aphidiinae) see separate key

4 Fore wing with triangular radial cell (Figure 2A). Pronotum in lateral view extending posteriorly to the tegula. Body rarely metallic in color...................................................................................................CYNIPOIDEA (Figitidae: Charipinae) see separate key

— Fore wing without radial cell. Pronotum in lateral view separated from tegula. Body sometimes metallic in color. ........................................................5 (CHALCIDOIDEA)

5 Antennal club elongate, sausage-shaped, not divided into antennomeres. Fore wing without setae on disk (Figure 2E,F). ..................................................6 (Signiphoridae)

— Antennal club absent, or if present, never sausage-shaped, and divided into antennomeres. Fore wing with many setae on disk. ....................................................7

6 Fore tibial spur pectinate (comb-like); fore wing as in Figure 2E. ...............*Signiphora*

— Fore tibial spur simple; fore wing as Figure 2F. ...........................................*Chartocerus*

7 Fore wing with postmarginal vein (Figure 1E–I), occasionally short. .......................8

— Fore wing without postmarginal vein (Figure 1A–D). ...............................................12

8 Wing infuscate below marginal vein, postmarginal vein about as long as stigmal vein (Figure 1E). .........................................................................*Moranila* (Moranilidae)

— Wing hyaline, postmarginal vein clearly longer than stigma vein (Figure 1F–I)..........9

9 Stigmal vein with uncus narrow (Figure 1F). ....................................................*Asaphes*

— Stigmal vein with uncus enlarged (Figure 1G,I), or greatly enlarged (Figure 1H). ..10 (Pteromalidae)

10 Metasoma in lateral view strongly convex dorsally; antennae inserted above center of face. .....................................................................................................................................................................................................................................................................*Euneura*

— Metasoma flat or slightly convex; antennae inserted near mouth. ..........................11

11 Stigmal vein with uncus greatly enlarged, wing base densely setose (Figure 1H). ....................................................................................................................................*Coruna*

— Stigmal vein with uncus moderately enlarged, wing base sparsely setose (Figure 1I). ...........................................................................................................................................................................................................................................................................*Pachyneuron*

12 Marginal vein much longer than stigmal vein (Figure 2B,C). ...................................13

— Marginal vein shorter than stigmal vein (Figure 1A,C) or about the same length. ......................................................................................................................18 (Encyrtidae)

13 Tarsi 4-segmented. ..................................................................................14 (Eulophidae)

— Tarsi 5-segmented. ................................................................................15 (Aphelinidae)

14 Postmarginal vein absent (Figure 2G). .........................................................*Tetrastichus*

— Postmarginal vein present (Figure 2H). ...........................................................................................................................................................................................................*Pediobius*

15 Antenna with 7 or 8 antennomeres; fore wing without linea calva (Figure 2C). ..........................................................................................................................................*Encarsia*

— Antenna with 6 or fewer antennomeres; fore wing with linea calva (Figure 2B). ...16

16 Fore wing with patterned areas of dark and pale infuscation (Figure 2B). ...*Marietta*

— Fore wing hyaline, rarely with an isolated area of infuscation (Figure 2D). ...........17

17 Tarsal claws equal in length. ............................................................................*Aphelinus*

— Tarsal claws unequal in length. ..................................................................*Protaphelinus*

18 Fore wing with dark and pale areas (Figure 1B). ................................*Cerapteroceroides*

— Fore wing hyaline. ............................................................................................................19

19 Fore wing with two lines of robust setae below submarginal vein (Figure 1D). ..................................................................................................................................*Tassonia*

— Fore wing with areas of dense, scattered setae below submarginal vein. ................20

20 Stigmal vein longer than marginal vein (Figure 1A). ..............................*Bothriothorax*

— Stigmal vein shorter than, or nearly as long as, marginal vein (Figure 1C). ..........................................................................................................................*Syrphophagus*

#### 3.2.1. Key to Charipinae Genera Associated with Aphids

1 Mesopleuron without mesopleural triangle (Figure 3C). Head and mesosoma with fine reticulate sculpture. Nearctic. ....................................................................*Lytoxysta*

— Mesopleuron with mesopleural triangle (Figure 3A,C). Head and mesosoma not sculptured. Cosmopolitan. ..............................................................................................2

2 Mesopleuron ventrally with horizontal sulcus (Figure 3A). .................*Phaenoglyphis*

— Mesopleuron without horizontal sulcus (Figure 3B). ....................................*Alloxysta*

#### 3.2.2. Key to the Genera of Aphidiinae of Economic Importance (Females)

1 Fore wing venation with seven closed cells; vein 3RSb reaching R1 vein at tip of wing margin, marginal cell closed (Figure 4G). ..............................................*Ephedrus*

— Fore wing venation with at most four closed cells or fewer; vein 3RSb not reaching the wing margin; marginal cell open (Figure 4A–F,H–J and Figure 5A–F). .......................2

2 Fore wing RS+M vein present (Figure 5D). Notauli well developed and complete (Figure 6C). ................................................................................................................*Praon*

— Fore wing RS+M vein absent (Figure 4A–F,H–J and Figure 5A–C,E,F). Notauli developed only in anterior part of mesonotum (Figure 6A,D) or absent (Figure 6B)..................3

3 Ovipositor sheaths cup-shaped (Figure 7F) or plowshare-shaped (Figure 7C,H–J,O). ..............................................................................................................................................4

— Ovipositor sheath of different shapes, triangular (Figure 7A,G), subovoid (Figure 7B,D,K), quadrangular (Figure 7E), spatulated (Figure 7L), slender (Figure 7M), acinaciform (Figure 7N). .................................................................................................8

4 Terminal metasomal sternum with a pair of prongs (Figure 7C,O). .........................5

— Terminal metasomal sternum without prongs (Figure 7F,H–J). ...............................6

5 Petiole with only primary (spiracular) tubercles (Figure 6S). ..........................*Trioxys*

— Petiole with both primary (spiracular) and secondary tubercles (Figure 6M). .........................................................................................................................................*Binodoxys*

6 Fore wing R1 distinctly longer than stigma; r & RS vein extending to level of tip of R1 vein, reaching close to the outer border of fore wing (Figure 4H). Ovipositor sheath elongated cup-shaped (Figure 7F). Petiole dorsally with a pair of strong carinae, diverging backwards (Figure 6N) or with crenulated dorsolateral carinae (Figure 6O). ...........................................................................................................*Lipolexis*

— Fore wing R1 distinctly shorter than stigma; r & RS vein not reaching the end of R1 vein, stands far from the outer border of wing margin (Figure 4J and Figure 5A,B). Ovipositor sheath of plowshare shaped (Figure 7H–J). Petiole dorsally with different patterns, striated or reticulated (Figure 6P–R). ............................................................7

7 Antenna moniliform. Fore wing stigma widely triangular (Figure 4J). Propodeum irregularly areolated or reticulated (Figure 6H). Petiole short and subquadrate, at most 1.2× as long as wide at spiracles (Figure 7H). .................................*Monoctonia*

— Antenna filiform. Fore wing stigma narrowly triangular (Figure 5A–B). Propodeum regularly areolate (Figure 6I). Petiole elongated, more than 1.5× as long as wide at spiracles (Figure 7I,J). ....................................................................................*Monoctonus*

8 Ovipositor sheath triangular, sharply pointed at tip (Figure 7A,G). ........................9

— Ovipositor sheath of different shapes, bluntly truncated or rounded at tip or upcurved, subovoid (Figure 7B,D,K), quadrangular (Figure 7E), slender (Figure 7M), acinaciform (Figure 7N). ................................................................................................10

9 Fore wing M+m-cu vein incomplete, r and r-m veins distinct (Figure 4I). *Lysiphlebus*

— Fore wing M+m-cu and r and r-m veins absent (Figure 4A). ........................*Adialytus*

10 Propodeum with very narrow (Figure 6F) to small central areola (Figure 6E). .....11

— Propodeum with a wide central pentagonal areola (Figure 6J), with anterolateral and central carinae (Figure 6G,K), or smooth, with only slight depression in the posterior part (Figure 6L). ..............................................................................................12

11 Fore wing r-m vein present, M+m-cu complete (Figure 4B,C) or reduced in anterior part. ........................................................................................................................*Aphidius*

— Fore wing r-m and M+m-cu veins absent (Figure 4E). ................................*Diaeretiella*

12 Propodeum smooth (Figure 6L). Fore wing stigma isosceles triangular in shape (Figure 5F). Head and thorax with reticulated microsculpture. ...........*Xenostigmus*

— Propodeum carinated (Figure 6G,J,K). Fore wing stigma scalene triangular in shape (Figure 5C). Head and thorax smooth. .........................................................................13

13 Fore wing M+m-cu and r-m veins absent (Figure 4F). Notauli absent (Figure 6B). Ovipositor sheath stout, subquadrate (Figure 6F). .........................................*Diaeretus*

— Fore wing M+m-cu and r-m veins present (Figure 5C). Notauli present in anterior part of mesonotum. Ovipositor sheath elongated in different shapes (Figure 7K–N).....................................................................................................................................*Pauesia*

## 4. Discussion

The bulk of the present work is devoted to an illustrated key for the identification of 35 genera from seven families belonging to three superfamilies of Hymenoptera, which are primarily found in agricultural ecosystems around the world. The whole assemblage of the parasitoid wasps associated with aphids belong to various superfamilies and families, and there are clear intrinsic differences in their behaviour and regulatory effects (positive and negative) on the population of aphids. At least some of the primary parasitoids belonging to these genera are significantly important for the biological control of aphids [33]. Based on a wealth of empirical evidence, wasps from the subfamily Aphidiinae (family Braconidae) and family Aphelinidae (except a few hyperparasioid species—[17]) exhibit a high level of host specialization and have scientifically demonstrated impacts on controlling aphid populations [33]. Seven other families (Megaspilidae, Encyrtidae, Eulophidae, Moranilidae, Pteromalidae, Signiphoridae, Figitidae) are all hyperparasitoids [11,34,40,43,53,58,60]. Current taxonomic and ecological knowledge on hyperparasitoids is primarily focused on genera belonging to the families Encyrtidae, Pteromalidae, and Figitidae, which exhibit the highest abundance in various ecosystems. Consequently, their potentially destructive effects should never be overlooked in biological control programs at both limited levels and large-scale trans-regional projects in both the short and long term [40,60].

Species-level identification requires a focus on existing keys for each of these genera, and depending on the study region globally, it may lead to the discovery of parasitoid species that have previously been unknown [61].

Climatic and biogeographical differences, as well as the historical context of activities in natural and agro-ecosystems in various regions, determine the composition of different insect species associated with these habitats [62,63,64,65]. It is expected that similar species or a specific range of aphids are active on the same plants [66]; however, this is often not the case for the associated parasitoids [67,68]. Apart from ecological and behavioural effects, the characteristics of each type of agroecosystem directly affect the species composition of established parasitoids within it [69]. Nevertheless, at any given time, various species of parasitoids may be present in a single agroecosystem, each with variable populations that can have complex and sometimes pivotal effects on pest aphid populations [33,70]. Therefore, in addition to gaining sufficient knowledge about the characteristics of each habitat, having a comprehensive identification key for recognizing all taxa that may be associated with aphids is essential. This approach provides access to relevant research and operational records concerning biological control of pest aphids in a comprehensive (rather than regional) manner.

Our expectations from agroecosystems dictate the types of activities implemented for better plant growth and higher-quality crop production. These activities strongly determine the species composition of parasitoids residing in these habitats [71,72,73]; although they often lead to reduced species diversity, changing consumption patterns in society and markets may result in the emergence and increased activity of parasitoid or hyperparasitoid species that previously had very low and undetectable populations. Like many other pests, the introduction and spread of various economically important [74] or indifferent [75,76] aphid species through the transfer of plant materials (such as seedlings and commercial saplings) indicate a continuous invasion and expansion of damage caused by species that typically do not inflict significant harm in their native habitats. Many of the aphid-associated genera included in this identification key comprise species whose distribution is limited to countries involved in trade routes e.g., [55,77,78,79], and particularly hyperparasitoid species should be closely monitored in quarantine programs.

This work aimed to provide the scientific community with a proper visual tool to correctly identify the hymenoptera aphid parasitoids, at least to the genus level. Advances in integrative taxonomy, combining morphological and molecular approaches, have significantly improved species delimitation, aiding in the identification of cryptic species and host-parasitoid associations. Future research could be focused on refining taxonomic classifications and elucidating trophic interactions, above all from families whose knowledge is still pending a thorough investigation. In addition to direct parasitoidism of aphids, it is important to consider the assemblage of parasitoids that target aphid predators, such as chrysopids, coccinellids, and syrphids. Some species of Chalcidoidea, among others, have been reported to parasitize these predatory groups, influencing their population dynamics and, consequently, the overall aphid control efficiency. While this study focuses on the primary interactions between aphids and their parasitoids, acknowledging these additional trophic relationships provides a broader perspective on the complexity of aphid suppression in natural and agricultural ecosystems.

## Data Availability

Chalcidoidea host data are currently publicly available from https://ucd.chalcid.org hosted by TaxonWorks (accessed on 29 November 2024).
